# Cardiorespiratory fitness and development of childhood cardiovascular risk: The EXAMIN YOUTH follow-up study

**DOI:** 10.3389/fphys.2023.1243434

**Published:** 2023-08-23

**Authors:** Christoph Hauser, Eric Lichtenstein, Lukas Nebiker, Lukas Streese, Sabrina Köchli, Denis Infanger, Oliver Faude, Henner Hanssen

**Affiliations:** Department of Sport, Exercise and Health, Medical Faculty, University of Basel, Basel, Switzerland

**Keywords:** cardiorespiratory fitness, childhood cardiovascular health, retinal vessel diameters, pulse wave velocity, primary prevention

## Abstract

**Background:** Obesity- and hypertension-related cardiovascular (CV) risk has been shown to originate in childhood. Higher body mass index (BMI) and blood pressure (BP) have been associated with increased large artery stiffness and a lower microvascular arteriolar-to-venular diameter ratio (AVR) in children. This study aimed to investigate the association of cardiorespiratory fitness (CRF) with development of BMI, BP and vascular health during childhood.

**Methods:** In our prospective cohort study, 1,171 children aged 6–8 years were screened for CRF, BMI, BP, retinal vessel diameters and pulse wave velocity using standardized protocols. Endurance capacity was assessed by 20 m shuttle run test. After 4 years, all parameters were assessed in 664 children using the same protocols.

**Results:** Children with a higher CRF at baseline developed a significantly lower BMI (β [95% CI] −0.09 [−0.11 to −0.06] kg/m^2^, *p* < 0.001), a lower systolic BP (β [95% CI] −0.09 [−0.15 to −0.03] mmHg, *p* = 0.004) and a higher AVR (β [95% CI] 0.0004 [0.00004 to 0.0007] units, *p* = 0.027) after 4 years. The indirect association of CRF with development of retinal arteriolar diameters was mediated by changes in BMI.

**Conclusion:** Our results identify CRF as a key modulator for the risk trajectories of BMI, BP and microvascular health in children. Obesity-related CV risk has been shown to track into adulthood, and achieving higher CRF levels in children may help counteract the development of CV risk and disease not only in pediatric populations, but may also help reduce the burden of CVD in adulthood.

**Registration:** http://www.clinicaltrials.gov/ (NCT02853747).

## Introduction

Cardiovascular disease (CVD) remains a major cause of rising healthcare costs and premature mortality. Obesity and hypertension are among the main risk factors for the development and progression of CVD and cardiovascular (CV) mortality (Stanaway et al., 2018), and CVD has been shown to originate in childhood (Berenson, 2002; Thompson et al., 2007). Mean body mass index (BMI) and the prevalence of obesity dramatically increased worldwide in children and adolescents over the last 40 years (Abarca-Gómez et al., 2017). Nowadays, one out of four children in the Western world is overweight or obese (Ng et al., 2014), and every overweight child is at risk of becoming and overweight adult (Freedman et al., 2005) and suffering from adult CVD (Bibbins-Domingo et al., 2007; Graham et al., 2008; Twig et al., 2016). Furthermore, an increase in elevated blood pressure (BP) and hypertension among children and adolescents has been described (Yan et al., 2016), which is closely related to the increase in childhood overweight and obesity (Kit et al., 2015). An increment of 1 kg/m^2^ BMI accounts for 1.4 mmHg higher systolic blood pressure (SBP) in prepubertal children (Falaschetti et al., 2010). Both risk factors track from childhood into adulthood (Freedman et al., 2005; Oikonen et al., 2016), induce endothelial dysfunction, for example, and may lead to CV events later in life (Berenson, 2002; Bruyndonckx et al., 2013). Physical activity (PA) and cardiorespiratory fitness (CRF) play an important role in the prevention of CVD (Jeong et al., 2019). Objectively measured vigorous PA is positively associated with higher CRF(16), but CRF seems to relate more strongly to CVD risk factors than PA in healthy children and adolescents (Hurtig-Wennlöf et al., 2007; Ortega et al., 2008). CRF pertains to the physiological capability of the human body to take up oxygen through the pulmonary system, subsequently conveying it via the circulatory system to the specific muscles and thereby enables the supply of energy during periods of physical activity (Armstrong and Van Mechelen, 2017). In scientific practice, the 20-m shuttle run test (SRT) is a widely adopted field-based approach for assessing CRF. In the literature, some disparity exists concerning the validity coefficient between the 20 m SRT and the laboratory determined maximal oxygen uptake (VO_2_ max) established as “gold standard” (Leger and Lambert, 1982; Van Mechelen et al., 1986; Boreham et al., 1990; Liu et al., 1992; Mahoney, 1992; Barnett et al., 1993; McVeigh et al., 1995; Matsuzaka et al., 2004; Aziz et al., 2005; Chia et al., 2005). Nevertheless, an aggregated mean strong positive correlation of 0.62 has been documented between the 20 m SRT and VO_2_ max (Hamlin et al., 2014). Moreover, this correlation tends to increase when accounting for factors such as maturation and body fat mass (Hamlin et al., 2014). In fact, it does not directly quantify VO_2_ max but serves as a reliable estimation and effectively reflects an individual’s endurance capacity (Mayorga-Vega et al., 2015). Development of CRF during childhood and adolescence is highly individualized and influenced by morphological and physiological changes that occur with growth and maturation and is further affected by strength, agility, motor coordination and body composition (Ortega et al., 2008; Armstrong and Van Mechelen, 2017; Armstrong and Welsman, 2019). However, literature demonstrates that increasing CRF in children and adolescents through adequate training at high intensities levels is irrespective of age, gender or maturity status (Armstrong and Barker, 2011). Studies conducted with children and adolescents have demonstrated that individuals with a high level of CRF tend to exhibit significantly lower total and lower abdominal adiposity (González-Gross et al., 2003; Moreno et al., 2003; Ara et al., 2004; Ruiz et al., 2006; Lee and Arslanian, 2007; Ortega et al., 2007). This association has been consistently observed, even in overweight and obese children (Nassis et al., 2005). Furthermore, markers of total and abdominal adiposity have been linked to blood pressure and obesity has been ascribed the role of a mediator between CRF and BP (Rizzo et al., 2007; Pozuelo-Carrascosa et al., 2017). In general, having a higher CRF level has been linked to a more favorable CV profile among children and adolescents (Reed et al., 2005; Andersen et al., 2006; Mesa et al., 2006; Ruiz et al., 2006; Ruiz et al., 2007). Prospective studies have shown that adolescents’ CRF is inversely related with adult CVD risk factor profiles (Andersen et al., 2004; Ferreira et al., 2005).

Retinal vessel diameters and central pulse wave velocity (PWV) are non-invasive and valid biomarkers of cardiovascular risk in children and adults and represent two different sections of the vascular tree (Mattace-Raso et al., 2006; Liew et al., 2008; Mitchell et al., 2010; Gopinath et al., 2013). In adults arteriolar narrowing and venular widening have been associated with increased CV risk and incidence CVD across all age groups (Hanssen et al., 2022). Furthermore, an increase in central PWV by 1 m/s corresponds to a risk increase of 15% for CV mortality and all-cause mortality (Vlachopoulos et al., 2010). Higher BP and BMI as well as lower CRF have been associated with vascular changes in childhood and adolescence in the micro- and macrocirculation. In a previously published systematic review and meta-analysis, our research group demonstrated that children and adolescents with higher BMI have narrower central retinal arteriolar equivalents (CRAE) and wider central retinal venular equivalents (CRVE) (Köchli et al., 2018). In the same age cohort, children and adolescents with elevated BP had narrower arteriolar diameters (Köchli et al., 2018). Furthermore, higher BP and BMI are associated with higher central PWV in children, whereas higher CRF is associated with lower central PWV (Lona et al., 2022). On a pathophysiological level, retinal arteriolar narrowing represents microvascular dysfunction and/or structural remodeling, whereas venular dilatation has been linked with systemic inflammation (Hanssen et al., 2022). Higher PWV is considered an estimate of increased central arterial stiffness and has been linked with progressive elastin degradation and enhanced collagen deposition in the arterial wall. The functional and structural impairments of large artery wall integrity have been associated with incidence CV events and all-cause mortality in adults (Vlachopoulos et al., 2010).Follow-up studies on the development of CRF with microvascular health and large artery stiffness in children are scarce. At the cross-sectional level in children aged 6 to 8 years, higher CRF was associated with wider retinal arteriolar and narrower retinal venular diameters and a lower PWV (Imhof et al., 2016; Köchli et al., 2019a). In our current large-scale longitudinal follow-up study, we aimed to assess the association of CRF with development of BMI, BP and vascular health over 4 years. We further aimed to investigate whether CRF and changes in risk factors were associated with retinal microvascular health and large artery stiffness at follow-up.

## Materials and methods

The data that support the findings of this study are available from the corresponding author on reasonable request.

### Study design and participants

In 2016/17, baseline data were obtained from all elementary schools in Basel (Switzerland) and every child was provided the opportunity to take part in the examinations. The study included children between age six and eight at baseline who had parental consent for medical screening. Medical screening took place during regular class hours in the morning while the children were in a fasted state. Medical assessments focused on blood pressure and vascular health. Anthropometry and CRF assessments were mandatory for all children and were performed by trained scientific staff during physical education lessons instead of regular classes. Four years later, follow-up examinations were conducted under the same conditions. The results of the baseline analyses have previously been published (Köchli et al., 2019a). At the outset of the study, a total of 1,171 children underwent medical, CRF and anthropometric assessments during the period of 2016/17. Subsequently, during follow-up, complete data were available for 664 children out of the initial cohort. The study was approved by the Ethics Committee of Northwestern and Central Switzerland (EKNZ, No. 258/12) and registered on ClinicalTrials.gov (http://www.clinicaltrials.gov/: NCT02853747). The study adhered to the guidelines for good clinical practice and the Strengthening the Reporting of Observational Studies in Epidemiology statement (Association, 2001).

### Measurements

The same devices and standardized procedures were applied at baseline in 2016/17 and at follow-up in 2020/2021 to ensure standardization of individual changes over time. All measurements were performed by trained scientific stuff.

### Cardiorespiratory fitness

The 20-m shuttle run test (SRT) is a reliable and reproducible measure for maximal endurance capacity in children (Van Mechelen et al., 1986; Léger et al., 1988). Participants were instructed to run back and forth between two parallel lines 20 m apart, while keeping up with audio-based pacing signals. The test began at an initial running speed of 8 km/h and increased by 0.5 km/h every minute. The test concluded when the participants reached exhaustion or failed to reach the line twice in a row within a 2-m range. The number of laps achieved was used for further analysis.

### Retinal vessel diameters

Retinal vessel analysis was performed using a fundus camera (Topcon TRC NW) and analysis software (Visualis 3.0, iMEDOS Health GmbH, Jena, Germany). Two valid images of each eye, with the optic nerve head centered and at a 45° angle, were captured. Two experienced examiners semi-automatically evaluated retinal arteriolar and venular diameters (Vesselmap 2, Visualis, iMEDOS Health GmbH, Jena, Germany) within a range of 0.5 to 1-disc diameter from the edge of the optic nerve head as previously described (Köchli et al., 2019b; Streese et al., 2021). CRAE and CRVE were averaged applying the Parr-Hubbard formula, and the arteriolar-to-venular ratio (AVR) was calculated using CRAE and CRVE (Hubbard et al., 1999). Retinal vessel analysis is a computer-based, semi-automated tool with remarkable reproducibility. In our previous work, it demonstrated an intraclass correlation coefficient ranging from 0.90 to 0.95 and a coefficient of variation of 2% when applied in young children (Imhof et al., 2016). To ensure optimal standardization, the same vessels and vessel segments were marked using baseline assessment initial values as a reference for retinal analysis.

### Pulse wave velocity

To determine central pulse wave velocity, the oscillometric Mobil-O-Graph monitor (I.E.M. GmbH, Germany) was used. The method has been validated for children and shows good agreement with the conventional tonometric approach (central SBP: −2.0 ± 5.6 mmHg compared with the reference method) (Wassertheurer et al., 2010; Mynard et al., 2020). Appropriate cuff size was selected based on the upper arm’s circumference and applied to the participant’s left arm in a sitting position. After resting for 5 min, the device was calibrated using SBP. At least two measurements were taken, with a two-minute interval between each measurement. Each measurement was closely inspected for quality, erroneous values, and repeated if necessary to calculate the mean and standard deviation (SD) of at least two measurements with good quality.

### Body composition

Participants’ height was measured while standing upright without shoes, using a stadiometer (Seca, Basel, Switzerland). Body weight was measured with a calibrated weight scale (InBody 170, Biospace device, InBody Co. in Seoul, Korea), while the participants wore light sportswear and were barefoot. Body mass index was computed by dividing weight in kilograms by the square of height in meters. To categorize BMI values, the age- and sex-specific reference values provided by Cole et al. (2000) were applied. Children with a BMI below the 85th percentile were considered to have normal weight, those between the 85th and 95th percentiles were classified as overweight, and those above the 95th percentile were categorized as obese.

### Blood pressure

Blood pressure assessments were conducted while participants were in a seated position after a 5-min rest period. The measurements were taken using either the automated oscillometric device Oscilomate 9002 (Oscillomate; CAS Medical Systems, Branford, CT) or the Mindray VS-900 (Mindray Bio-Medical Electronics Co., Ltd., Shenzhen, China). Both algorithms for measuring blood pressure have been validated in children (Alpert, 1996; Wong et al., 2006; Lang et al., 2014; Streese et al., 2022) and do not differ significantly from each other (Streese et al., 2022). Appropriate cuff size was selected based on individual’s upper arm circumference, following guidelines (National High Blood Pressure Education Program Working Group on High Blood Pressure in Children and Adolescents, 2004; Flynn et al., 2017). Five measurements were taken, with a 1-min rest period between each, and the mean of the three measurements with the smallest variation was used for further analysis. Systolic and diastolic BP were classified based on age- and gender-specific reference values from the German Health Interview and Examination Survey for Children and Adolescents, which takes into account individual height (Neuhauser et al., 2011). Children with a BP above the 90th percentile were classified as having elevated BP, and those above the 95th percentile were categorized as being in the hypertensive range.

### Statistical analysis

To describe the population characteristics, means and standard deviations (SD) were calculated for both baseline and follow-up data, and a t-test for paired samples was performed to compare the two. To evaluate potential selection bias, a t-test for independent samples was conducted between follow-up and lost-to-follow-up groups. To determine changes in population characteristics we have used a simple t-test to analyze differences from baseline to follow-up. Multiple imputation using chained equation (MICE) was performed to account for missing data of height, weight, BMI and SES (White et al., 2011). We imputed 50 datasets using predictive mean matching. Directed acyclic graphs (DAGs) were used to identify confounders necessary to minimize bias in estimates (Tennant et al., 2021). To investigate the association between cardiorespiratory fitness at baseline with BP, BMI, retinal vessel diameters and PWV at follow-up, a linear mixed regression model was applied, using schools and classes nested in schools as random effects adjusted for sex, SES at baseline and age at follow-up (Twisk, 2006; West et al., 2006). All variables, except for sex, were incorporated into the model as continuous variables. Distribution of variables were inspected *a priori* using histograms and assumptions for regression models were checked graphically using residual plots (Brown and Prescott, 2015). Additionally, we conducted path analysis to examine the direct and indirect effects of CRF at baseline on micro- and macrocirculation, as influenced by changes in BMI and BP (Ullman and Bentler, 2012). The regression analyses are presented with β coefficients and the corresponding 95% confidence intervals (CI). Marginal predicted means were used for graphic representation. Sample size and power calculation have been reported elsewhere (Lona et al., 2020). All tests were 2-sided, and the significance level was set at 0.05. All calculations were performed using Stata 15 (StataCorp, College Station, TX, United States).

## Results

### Populations characteristics

**FIGURE 1:**
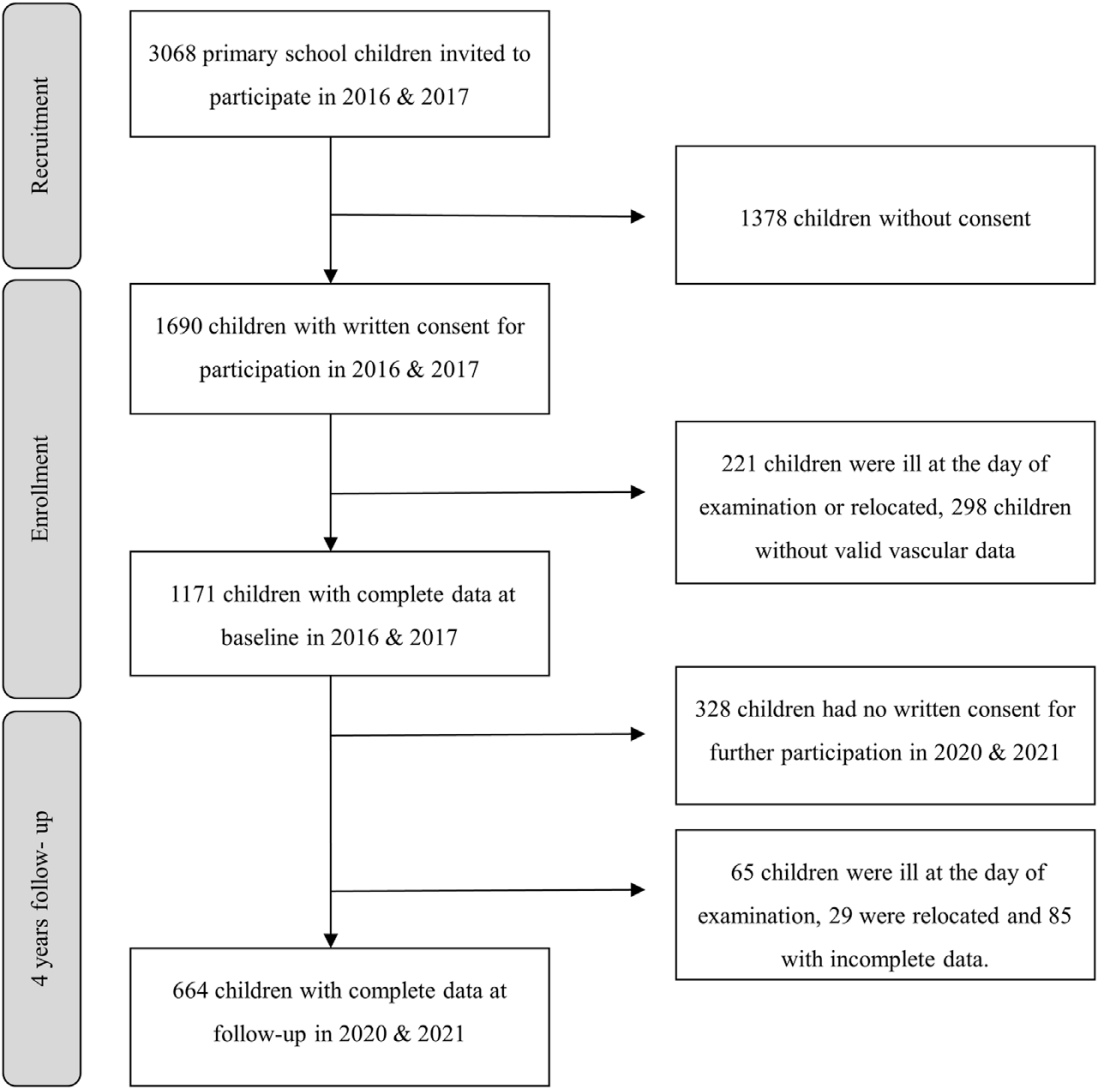
Flow-chart.

Initially, 1,171 **c**hildren underwent medical, CRF and anthropometric assessment in 2016/17, and among them, 664 had complete data at follow-up (Figure 1). Population characteristics are presented in absolute values and standard deviation (SD) for baseline, follow-up, and mean differences over time (Table 1). The follow-up group had significant higher CRF level (31.1 vs. 28.9 laps; *p* = 0.004) at baseline; however, we found no evidence for differences with respect to other population characteristics between the lost-to-follow-up group (36%). Baseline measurements revealed a prevalence of 10.5% for elevated SBP among children, with 14.8% falling into the hypertensive range. Moreover, 9.1% of the children exhibited elevated diastolic blood pressure (DBP), while 15% displayed DBP in the hypertensive range. The prevalence of overweight and obesity at baseline was 9.4% and 2.8% respectively. Over a span of 4 years, the children experienced increases in BMI (∆2.5 ± 2.1 kg/m^2^), SBP (∆5 ± 9.4 mmHg), and PWV (∆0.3 ± 0.3 m/s). Additionally, a statistically significant narrowing of CRAE (∆-7.2 ± 8.0 μm), CRVE (∆-1.4 ± 8.8 μm), and a decrease in AVR (∆-0.02 ± 0.04) were observed in the children at follow-up compared to their baseline measurements. It is worth noting that girls consistently exhibited wider CRAE and CRVE in comparison to boys at both time points. Furthermore, boys had statistically significantly higher CRF level than girls at both time points, as indicated by the data presented in Table 1.

**TABLE 1:** Population characteristics at baseline and follow-up.

Parameter	2016/2017			2020/2021			Difference		
	N	Mean	SD	N	Mean	SD	Mean	SD	*p*-value
Sex (female, %)	391	51.86							
Age, y	747	7.2	0.36	747	11.4	0.4	4.2	0.2	<0.001
BMI, kg/m^2^	736	15.7	2.0	544	18.2	3.4	2.5	2.1	<0.001
Male	347	15.8	2.1	258	18.4	3.6			
Female	379	15.7	2.1	286	18.0	3.2			
Height, cm	727	124.5	5.4	538	149.4	7.2	24.9	4.3	<0.001
Weight, kg	726	24.5	4.5	544	40.9	9.8	16.4	6.6	<0.001
CRF, laps	720	31.1	12.3	533	49.1	19.3	17.3	16.1	<0.001
Male	344	34.4*	13.1	254	55.1*	20.3			
Female	376	28.2*	10.8	279	43.5*	16.5			
SBP	749	104	7.8	749	109	9.8	5	9.4	<0.001
DBP	749	64	6.8	749	65	7.8	1	8.2	0.279
CRAE, µm	694	202.6	12.8	724	195.4	12.3	−7.2	8.0	<0.001
Male, µm	339	200.1*	12.4	346	192.7*	11.8			
Female, µm	335	205.0*	12.7	378	197.8*	12.3			
CRVE, µm	694	229.6	13.9	724	228.2	13.9	−1.4	8.8	<0.001
Male, µm	339	227.9*	14.1	346	226.1*	13.5			
Female, µm	355	231.3*	13.6	378	230.1*	14.1			
AVR	694	0.88	0.05	724	0.86	0.05	−0.02	0.04	<0.001
Male	339	0.87	0.05	346	0.85*	0.05			
Female	355	0.88	0.05	378	0.86*	0.05			
PWV, m/s	664	4.3	0.3	729	4.6	0.3	0.3	0.3	<0.001
Male, m/s	322	4.3	0.3	346	4.6	0.3			
Female, m/s	342	4.3	0.3	383	4.6	0.3			

AVR, arteriolar-to-venular diameter ration; BMI, body mass index; CRAE, central retinal arteriolar equivalent; CRF, cardiorespiratory fitness; CRVE, central retinal venular equivalent; DBP, diastolic blood pressure; PWV, pulse wave velocity; SBP, systolic blood pressure.*indicates a significant difference between sex.

### Cardiorespiratory fitness and development of risk factors

**FIGURE 2:**
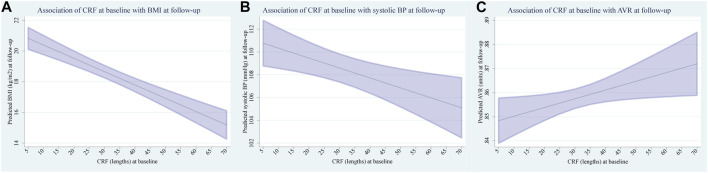
**(A)** Marginal predicted means of body mass index at follow-up based on cardiorespiratory fitness at baseline. **(B)** Marginal predicted means of systolic blood pressure at follow-up based on cardiorespiratory fitness at baseline. **(C)** Marginal predicted means of arteriolar-to-venular ratio at follow-up based on cardiorespiratory fitness at baseline.

The association between CRF at baseline and risk factors at follow-up are presented in Table 2. Across the whole population, children with a higher CRF at baseline developed a significantly lower BMI (β [95% CI] −0.09 [−0.11 to −0.06] kg/m^2^ decrease per additional lap in SRT, *p* < 0.001) at follow-up. The corresponding plot with marginal predicted means of BMI at follow-up, based on CRF at baseline, is shown in Figure 2A. Furthermore, children with a higher CRF at baseline developed significantly lower SBP (β [95% CI] −0.09 [−0.15 to −0.03] mmHg decrease per additional lap in SRT, *p* = 0.004) at follow-up (Figure 2B). We found little evidence for an association between CRF at baseline and DBP (β [95% CI] −0.03 [−0.08 to 0.02] mmHg decrease per additional lap in SRT, *p* = 0.233) at follow-up.

**TABLE 2:** Association of cardiorespiratory fitness at baseline with risk factors at follow-up.

Parameter	BMI at follow-up (kg/m^2^ decrease per additional lap in SRT)		SBP at follow-up (mmHg decrease per additional lap in SRT)		DBP at follow-up (mmHg decrease per additional lap in SRT)	
	B (95% CI)	B (95% CI)	B (95% CI)	*p*-value	B (95% CI)	*p*-value
CRF at baseline (laps in SR)*	−0.09 (−0.11 to −0.06)	<0.001	−0.09 (−0.15 to −0.03)	0.004	−0.03 (−0.08 to −0.02)	0.233

BMI, body mass index; CRF, cardiorespiratory fitness; DBP, diastolic blood pressure; SBP, systolic blood pressure; SR, shuttle run. *adjusted for sex, SES, at baseline and age at follow-up.

### Cardiorespiratory fitness and development of vascular health

The associations between baseline CRF and vascular health at follow-up are presented in Table 3. After adjustment for sex, SES at baseline and age at follow-up, children with a higher CRF at baseline developed a significantly higher AVR (β [95% CI] 0.0004 [0.00004 to 0.0007] units increase per additional lap in SRT, *p* = 0.027). The corresponding plot with marginal predicted means of AVR at follow-up, based on CRF at baseline, is shown in Figure 2C. We found no evidence for an association between baseline CRF with CRAE (β [95% CI] 0.01 [-0.07 to 0.08] μm increase per additional lap in SRT, *p* = 0.838) and CRVE (β [95% CI] −0.08 [−0.17 to 0.01] μm decrease per additional lap in SRT, *p* = 0.064) at follow-up. Additionally, our findings suggest limited support for an association between baseline CRF and PWV (β [95% CI] −0.001 [−0.003 to 0.001] m/s decrease per additional lap in SRT, *p* = 0.202) at follow-up.

**TABLE 3:** Association of cardiorespiratory fitness at baseline with vascular health at follow-up.

Parameter	AVR at follow-up (Units increase per additional lap in SRT)		CRVE at follow-up (µm decrease per additional lap in SRT)		CRAE at follow-up (µm increase per additional lap in SRT)		PWV at follow-up (m/s decrease per additional lap in SRT)	
	B (95% CI)	*p*-value	B (95% CI)	*p*-value	B (95% CI)	*p*-value	B (95% CI)	*p*-value
CRF at baseline (laps in SR)*	0.0004 (0.00004–0.0007)	0.027	−0.08 (−0.17 to 0.01)	0.064	0.01 (−0.07–0.08)	0.838	−0.001 (−0.003 to 0.001)	0.202

AVR, indicates arteriolar-to-venular diameter ration; CRAE, central retinal arteriolar equivalent; CRF, cardiorespiratory fitness; CRVE, central retinal venular equivalent; PWV, pulse wave velocity; SES, socioeconomic status; SR, shuttle run. *adjusted for sex, SES, at baseline and age at follow-up.

### Cardiorespiratory fitness, changes in risk factors and development of vascular health

**FIGURE 3:**
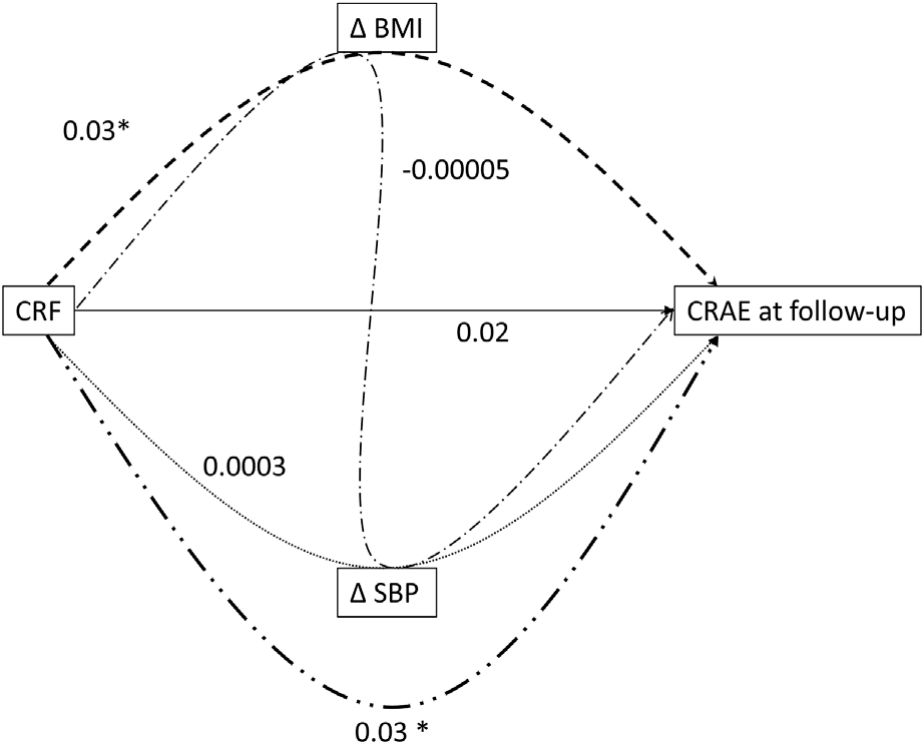
Direct and total indirect effects of cardiorespiratory fitness mediated by changes in body mass index and blood pressure on CRAE at follow-up.

Baseline CRF was not accompanied by direct significant changes in CRAE, CRVE and large pulse wave velocity at follow-up. However, analysis of the interrelation between CRF, ∆ BMI, ∆ SBP and CRAE at follow-up using simplified path diagram (Figure 3) revealed that a higher CRF level at baseline resulted in a less pronounced increase in BMI, which was associated with an increase in CRAE (β [95% CI] 0.03 [0.008 to 0.05] μm, *p* < 0.001). We found no evidence that higher CRF at baseline lead to a favorable change in SBP over the follow-up period and thus improved CRAE. We observed a significant total indirect effect of higher CRF on wider CRAE at follow-up (β [95% CI] 0.03 [0.01 to 0.05] μm per additional lap in SRT, *p* = 0.006). No significant direct and total indirect effects on CRVE and PWV were found (Supplement Material, Supplementary Figure S1; Supplementary Table S1).

## Discussion

As main findings, higher CRF at baseline was associated with lower BMI and lower SBP at follow-up. At the microvascular level, children with a higher CRF at baseline showed a significant higher AVR after 4 years. In other words, higher fitness levels were associated with improved retinal microvascular health after 4 years, mediated by BMI. Our results did not reveal a significant direct association between CRF and PWV as a marker of macrovascular health.

The results of our analysis imply that CRF is inversely associated with BMI progression in young children after adjustment for sex, SES and age. Our results are in line with results from a previously published review on longitudinal studies including 38 articles, which found an inverse association between higher CRF during childhood and adolescents and lower BMI later in life (Mintjens et al., 2018). The causal association between BMI and fitness or PA patterns in childhood is a subject of ongoing scientific debate (Bürgi et al., 2011; Hallal et al., 2012; Tanaka et al., 2018). Insufficient PA has been associated with a higher risk of weight gain in preschool children, but the reverse relationship is not well-supported (Bürgi et al., 2011). Additionally, established childhood obesity may impede the ability to engage in vigorous physical activities and adversely affect CRF performance (Goran et al., 2000). A positive correlation has been established between PA and CRF (Wagner et al., 2021). However, a considerable portion of children and adolescents fails to meet the recommended levels of 60 min at moderate to vigorous PA per day (Guthold et al., 2018; Guthold et al., 2020). Based on this premise, it can be hypothesized in a very simplified manner that the majority of children who engage in higher levels of PA may exhibit a significantly higher daily energy expenditure, consequently resulting in a more moderate increase in BMI over the investigation period.

After accounting for age, sex and SES, children with a lower CRF at the initial assessment exhibited higher SBP during the follow-up period and *vice versa*. These findings are consistent with previous cross-sectional investigations that have demonstrated a significant inverse association between CRF and SBP as well as DBP (Klasson-Heggebø et al., 2006; Nielsen and Andersen, 2003). Moreover, various longitudinal studies in children and adolescents have produced comparable findings (Juhola et al., 2012; Franklin and Pierce, 2014; Agostinis-Sobrinho et al., 2018). Nonetheless, when examining a 20-year follow-up period from adolescence, similar patterns were previously observed in the relationship between CRF and BP, although these trends did not reach statistical significance (Kelly et al., 2015). Moreover, the impact of BMI on BP should also take into account. There is evidence suggesting that BMI serves as a mediator in the relationship between CRF and BP. Pozuelo-Carrarascosa and colleagues conducted a study involving 1,604 school children aged 4–7 years. The results indicated that BP was significantly higher in children with poor fitness and overweight. Additionally, BMI acted as a mediator in the association between CRF and mean arterial pressure (Pozuelo-Carrascosa et al., 2017). Similar results were found by Ruiz et al. (2007) where females with hypertension had significantly higher fatness and lower CRF compared to females with normal BP. The elevation of BP is influenced by multiple factors and characterized by intricate interactions. It is known with a high level of evidence that CRF exerts positive effects on various physiological levels, including vascular, hormonal, and neuronal pathways. Increased CRF levels are associated with reduced vascular resistance, improved endothelial function and higher shear stress exposure, decreased oxidative stress and sympathetic activity, and improved insulin sensitivity, all of which contribute to the overall positive effect on BP regulation (Diaz and Shimbo, 2013).

However, CRF, BP, and BMI seems to be interrelated and contribute to the development of CVD. In adults, CRF, PA, and BMI have been associated with CVD, CV mortality and morbidity (Hubert et al., 1983; Paffenbarger et al., 1986; Blair et al., 1989; Manson et al., 1995; Blair et al., 1996; Shaper et al., 1997; Lee et al., 1999). The Aerobic Center Longitudinal Study performed in adults concluded that low physical fitness resulted in a greater risk of mortality than fatness, whereas fitness diminished the impact of fatness on mortality (Blair et al., 1989; Lee et al., 1998). This “fat but fit” paradigm has been less studied in children. Pozuelo-Carrascosa et al. (2023) have found similar results, albeit without hard endpoints, in 312 children aged 9–12 years. Their results indicate that “fat-fit” and “unfit-fit” children had better levels of high-density lipoprotein cholesterol, triglycerides, insulin levels, vigorous PA amount and metabolic syndrome index than their peers in the “fat-unfit” and “unfit-unfit” categories. Furthermore, BMI acts as a mediator between CRF and BP. (Pozuelo-Carrascosa et al., 2017). In our examinations we have used 20 m SRT to investigate endurance capacity. Even though BMI does not directly influence VO_2_ max (Goran et al., 2000), it can significantly diminish performance during the 20 m SRT, where body weight and especially inactive fat mass may become constraining factors (Welsman and Armstrong, 2019). Hence, it is plausible that the presence of overweight or obesity at the initial assessment could have exerted an influence on the performance in the 20 m SRT and thus on BP progression.

In our cohort, prepubertal children with higher CRF levels at baseline exhibited a significant higher AVR after 4 years. This demonstrates the predictive value of higher CRF with improved microvascular function during childhood development. There was a tendency for CRF to be associated with wider CRAE and narrower CRVE without reaching significance for the single parameters. CRF was not associated with development of arterial stiffness over the 4 years. Previous cross-sectional studies have reported association between higher CRF levels and narrower venular diameters, higher arteriolar-to-venular ratio, and lower large artery pulse wave velocity (Imhof et al., 2016; Lona et al., 2022). Our results demonstrate that the relationship between CRF and CV risk as well as vascular health changes during childhood development. The initial level of CRF appears to relate best to development microvascular health during childhood rather than large artery stiffness. It is important to realize that retinal microvascular diameters and large artery PWV are indicative of different segments within the vascular tree. Retinal microvascular imaging offers a distinct and non-invasive approach to evaluate the microcirculation and resistance vessels in the human body, providing unique insights into microvascular health (Hanssen et al., 2022). On the other hand, central PWV serves as a reliable measure for assessing large artery wall integrity and macrovascular health (Wang et al., 2008). As previously shown, the adverse progression of both biomarkers is associated with an elevated risk of hypertension, stroke, CV morbidity and mortality in adults (Ikram et al., 2006; Ikram et al., 2006; McGeechan et al., 2009; Vlachopoulos et al., 2010). However, child development is a multifaceted and intricate process with short-term risk exposure in the early stages of life. In the light of our findings, it is evident that higher CRF is associated with improvement of microvascular health during childhood development and less so with changes in large artery stiffness.

While a direct effect of CRF at baseline on CRAE and CRVE and large artery PWV was not established, our study revealed a significant total indirect effect of higher CRF on CRAE and AVR, mediated through changes in BMI. A meta-analysis has provided evidence of an association between increased BMI and narrower arteriolar and wider venular diameters, which is likely influenced by obesity-related inflammatory processes (Wong et al., 2006; Boillot et al., 2013). Studies conducted in adults have revealed that individuals with obesity exhibit higher levels of vasoconstrictor molecules such as endothelin-1, angiotensin-II, and various arachidonic acid metabolites (Cardillo et al., 2002; Stepp et al., 2007). Nitric oxide (NO) serves as a crucial local vasodilator, and its reduced bioavailability appears to correlate with higher BMI levels (Stapleton et al., 2008). Higher CRF, in addition to its positive effects on various physiological pathways, holds the potential to mitigate inflammation and enhance NO bioavailability, thereby improving endothelial function (Diaz and Shimbo, 2013). In children with higher CRF, NO bioavailability may be enhanced and, on the other hand, inflammatory processes may be diminished, offering a potential explanation for the effects of higher CRF on microvascular health mediated through BMI. One possible explanation for the lack of association observed at the macrovascular level may be attributed to the inherent structural properties of the macrocirculation, which may be relatively more inert compared to the microcirculation. An increase in PWV is closely linked to structural alterations, including collagen deposition and elastin degradation, which may require a longer duration to manifest noticeable changes. Processes of macrovascular remodeling are likely to be more gradual, potentially necessitating longer-term exposure to lifestyle changes in order to observe significant alterations in PWV.

Our Study has some limitations. The assessment of CRF in our study was conducted using the 20 m SRT due to practical considerations and the school setting, which made spiroergometry impractical. Nonetheless, the 20 m SRT is a reliable and reproducible method for estimating maximal endurance capacity in children (Van Mechelen et al., 1986; Léger et al., 1988). Effect sizes in our cohort of otherwise healthy young children appear small and their clinical relevance for development of CV risk and disease development in adulthood need to be investigated in future studies. Our baseline investigations did not include dietary assessment, preventing us from capturing changes in diet over the course of the study. It is important to note that our study was conducted within a predominantly Caucasian population, which limits the generalizability of our results to other ethnic groups. Furthermore, the progress of our follow-up research was disrupted by the COVID-19 pandemic. The imposed restrictions, including temporary school closures and limitations in the built environment, may had an impact on physical activity patterns and overall wellbeing, potentially influencing our findings. However, it is worth noting that the prevalence of SARS-CoV-2 infection among Swiss schoolchildren remained low, even during periods of high incidence in the general population, with a low prevalence of asymptomatic cases (Kriemler et al., 2021). Hence, it is unlikely that direct effects of SARS-CoV-2 infection influenced the collected data.

## Conclusion

The findings of our study indicate the potential predictive value of baseline CRF in relation to the subsequent development of BMI and SBP as well as microvascular health. Whereby a phenotype characterized by favorable body composition and maximal endurance capacity may represent an optimal combination in terms of development of CV risk factors and vascular health. These results may hold significant scientific implications, highlighting the association between CRF and the trajectory of BMI, blood pressure and microvascular health in children. Nonetheless, the clinical relevance of our findings for the development of CV risk and disease later in life still needs to be established in future long-term follow-up studies from childhood into adulthood. We would like to underscore the potential and significance of promoting exercise to achieve higher CRF levels during childhood as a primary prevention strategy in order to mitigate excessive weight gain and elevated BP in early childhood. Long-term, exercise interventions with a focus on increasing CRF have the potential to lower childhood BMI and blood pressure. Most importantly, obesity-related CV risk tracks into adulthood (Freedman et al., 2005; Yang et al., 2006; Baker et al., 2007; Oikonen et al., 2016; Twig et al., 2016), and achieving higher CRF levels in children may help counteract the development of CVD not only in pediatric populations but may also reduce the burden of CVD in adulthood.

## Data Availability

The raw data supporting the conclusion of this article will be made available by the authors, without undue reservation.
