# Supplementary material for: Cardiorespiratory fitness and development of childhood cardiovascular risk: The EXAMIN YOUTH follow-up study
Source: Front Physiol. 2023 Aug 23;14:1243434. doi: 10.3389/fphys.2023.1243434 (PMC10482095; doi:10.3389/fphys.2023.1243434)

**Table S1. Direct and total indirect effects of cardiorespiratory fitness mediated by changes in body mass index and blood pressure on**

**vascular health at follow-up**

| Predictor | Mediator | CRAE at Follow-up (µm Change Per Unit Increase in CRF)* | |
| --- | --- | --- | --- |
|  |  | B (95% CI) | P Value |
| CRF | ∆ BMI | 0.03 (0.008 to 0.05) | 0.008 |
| CRF | ∆ SBP | -0.00005 (-0.009 to 0.0008) | 0.910 |
| CRF | ∆ BMI & ∆ SBP | 0.0003 (-0.004 to 0.005) | 0.901 |
| CRF | total indirect effect | 0.03 (0.01 to 0.05) | 0.006 |
| Predictor | Mediator | CRVE at Follow-up (µm Change Per Unit Increase)* | |
|  |  | B (95% CI) | P Value |
| CRF | ∆ BMI | -0.02 (-0.4 to 0.01) | 0.178 |
| CRF | ∆ SBP | 0.0002 (-0.001 to 0.002) | 0.825 |
| CRF | ∆ BMI & ∆ SBP | -0.001 (-0.006 to 0.004) | 0.709 |
| CRF | total indirect effect | -0.02 (-0.04 to 0.006) | 0.140 |
| Predictor | Mediator | AVR at Follow-up (Units Change Per Unit Increase)* | |
|  |  | B (95% CI) | P Value |
| CRF | ∆ BMI | 0.0002 (0.00009 to 0.0003) | 0.001 |
| CRF | ∆ SBP | -9.76e^-07^ (-7.91e^-06^ to 5.96e^-06^) | 0.783 |
| CRF | ∆ BMI & ∆ SBP | 4.67e^-06^ (-0.00002 to 0.00003) | 0.650 |
| CRF | total indirect effect | 0.0002 (0.0001 to 0.0003) | <0.001 |
| Predictor | Mediator | PWV at Follow-up (m/s Change Per Unit Increase)* | |
|  |  | B (95% CI) | P Value |
| CRF | ∆ BMI | -0.0003 (-0.0008 to 0.0001) | 0.159 |
| CRF | ∆ SBP | 0.00004 (-0.0002 to 0.0003) | 0.777 |
| CRF | ∆ BMI & ∆ SBP | -0.0002 (-0.0004 to -0.00007) | 0.004 |
| CRF | total indirect effect | -0.0005 (-0.001 to 0.00003) | 0.062 |

AVR indicates arteriolar-to-venular diameter ration; BMI, body mass index; CRAE, central retinal arteriolar equivalent; CRF, cardiorespiratory fitness; CRVE, central retinal venular equivalent; PWV, pulse wave velocity; SBP, systolic blood pressure *adjusted for sex and age

**Figure S1. Direct and total indirect effects of cardiorespiratory fitness mediated by changes in body mass index and blood pressure on**

**AVR at follow-up**


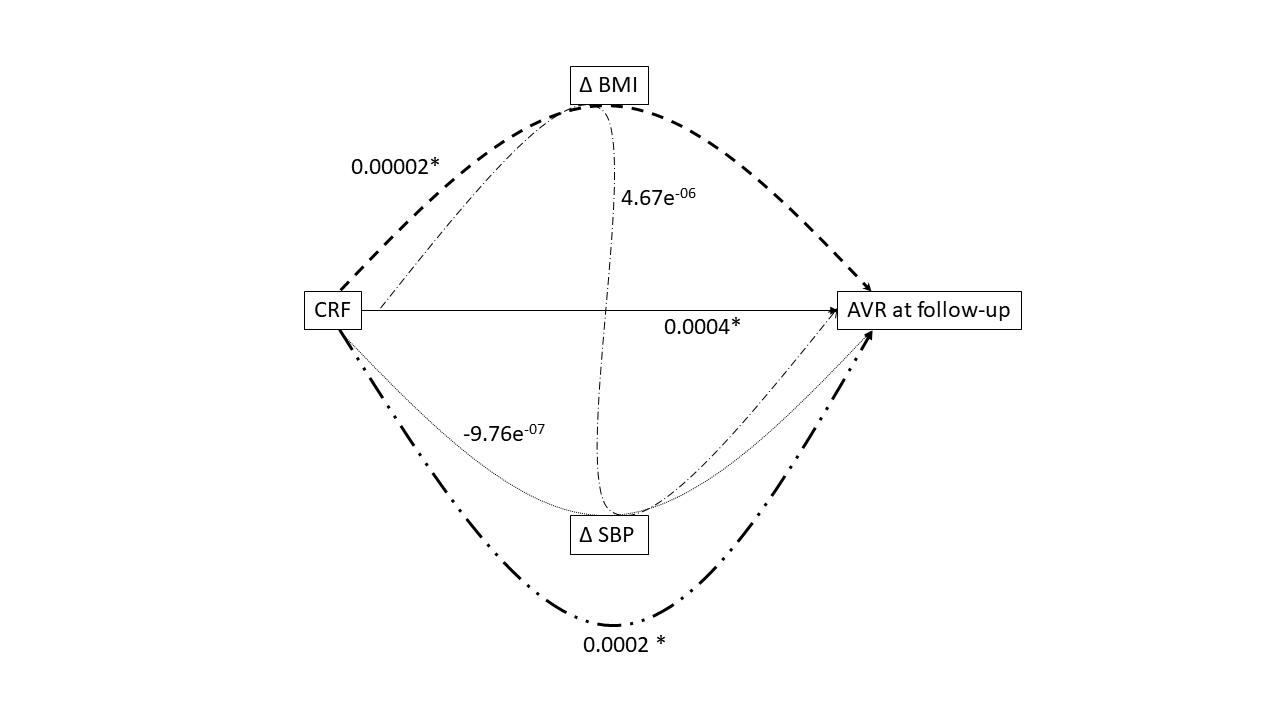

Supplement: Supplementary file 1 [file DataSheet1.docx]
